# Role of prophylactic coronary revascularisation in improving cardiovascular outcomes during non-cardiac surgery: A narrative review

**DOI:** 10.1007/s12471-016-0871-1

**Published:** 2016-08-18

**Authors:** T. Rahat, T. Nguyen, F. Latif

**Affiliations:** 1 University of Maryland Medical Center, Baltimore, MD USA; 2 Indiana University School of Medicine, Community Healthcare System, St Mary Medical Center, Hobart, Indiana USA; 3 University of Oklahoma Health Sciences Center & Veterans’ Affairs Medical Center, Oklahoma City, OK USA

**Keywords:** Coronary stenting, Perioperative, Revascularisation

## Abstract

Coronary revascularisation has been a topic of debate for over three decades in patients undergoing high-risk non-cardiac surgery. The paradigm shifted from routine coronary angiography toward stress test guided decision-making based on larger randomised trials. However, this paradigm is challenged by relatively newer data where routine coronary angiography and revascularisation is shown to improve perioperative cardiovascular outcomes. We review major studies performed over a long period including more contemporary data with regard to the 2014 American College of Cardiology/American Heart Association as well as 2014 European Society of Cardiology guideline on perioperative cardiovascular evaluation of patients undergoing non-cardiac surgery.

## Introduction

Cardiac risk stratification before any non-cardiac surgery is a common reason for a cardiology consultation. Despite the fact that current American College of Cardiology/American Heart Association (ACC/AHA) guidelines on perioperative cardiac evaluation indicate that coronary revascularisation before non-cardiac surgery should generally be reserved for patients who have an indication for it independent of the surgery, many more patients undergo coronary revascularisation in the preoperative setting, who would not undergo revascularisation otherwise [1]. These guidelines recommend the use of risk factor indices (such as the Revised Cardiac Risk Index (RCRI) and National Surgical Quality Improvement Program (NSQIP) risk calculator) and functional capacity of individual patients to guide further testing in preoperative evaluation in patients at elevated risk (>1 %) of perioperative major adverse cardiac events during planned non-cardiac surgery. On the other hand, European Society of Cardiology (ESC) guidelines recommend avoiding non-invasive testing in stable patients who have undergone coronary artery bypass graft (CABG) surgery within the last six years, based on the data from the Coronary Artery Surgery study [2, 3]. Based on various observational studies including the landmark study by Hertzer et al. in 1984, coronary revascularisation was associated with improved perioperative cardiovascular outcomes during non-cardiac surgery [4–8]. As stress testing techniques were refined, further studies evaluated a stress test-guided approach to risk stratify these patients to avoid the risks associated with invasive angiography [9, 10]. As a result, over the years, most routine coronary angiography and revascularisation procedures were supplanted by stress test-guided procedures to a variable degree. However, despite their shortcomings relatively newer trials have shown conflicting results with the use of routine angiography [11, 12]. Nevertheless, a debate still exists regarding the interpretation and applicability of information derived from these trials, and many physicians still utilise individual practice patterns, rather than an evidence-based approach. The discordance between opinions of two cardiologists can be as high as 54 % [13]. Part of the reason could be lack of incorporation of some newer trial data into the guidelines. The purpose of this review is to delineate the pathophysiological basis of perioperative myocardial ischaemia as well as review contemporary studies on perioperative assessment with particular emphasis on revascularisation vis-a-vis the current ACC/AHA guidelines.

## Pathophysiology of perioperative myocardial infarction

### Contributing factors

The pathophysiology of perioperative myocardial infarction (MI) is multifactorial. Surgery can induce a stress response in the body, with the release of pro-inflammatory and pro-thrombotic mediators that may predispose to platelet aggregation, plaque rupture, haemorrhage and subsequent infarction [14–16]. In addition, intraoperative hypotension due to fluid shifts, blood loss anaemia, pain and tachycardia can induce oxygen demand-supply mismatch in patients with fixed perfusion defects [14, 15]. Perioperative pain triggers sympathetic pathways, which could also produce myocardial ischaemia, as evidenced by a study showing reduction in ischaemia with the use of morphine [17]. Finally, based on ‘normal’ coronary angiograms prior to surgery in patients with perioperative MI, the possibility of transient coronary thrombosis and vasospasm also exists [18].

### Impact of ischaemia duration

Prolonged subendocardial ischaemia, as determined by episodes of prolonged ST-segment depression, has been associated with a vast majority of perioperative myocardial events [14–16, 19]. These were exclusively non-ST-segment elevation MIs. In addition, the timing of preoperative MI seems to be greatest in the period immediately after surgery and emergence from anaesthesia [14–16, 19]. Most troponin elevations occur within the first 12–24 hours after surgery [20]. This correlates with the period where tachycardia, alterations in blood pressure, pain and catecholamine response tends to be highest. A study involving vascular surgery patients showed reduced incidence of myocardial ischaemia if the heart rate was controlled strictly in the first 48 hours following surgery [21]. In a dog model with fixed coronary stenosis but without ischaemia at rest, sustained tachycardia triggered MI despite anticoagulation [22].

### Mechanism of perioperative MI

In an autopsy study on 26 patients with fatal perioperative MI, plaque rupture was discovered in half of the patients [23]. In a recent study involving 66 patients with perioperative MI, analysis of pre- and post-surgery coronary angiograms showed 54.5 % of infarctions to be from demand ischaemia, 25.8 % from thrombotic, and 19.7 % from non-obstructive coronary artery disease without any identifiable culprit lesion [24]. Another study reported by Dawood and colleagues showed perioperative MI to occur predominantly in patients with left main and triple-vessel disease [25]. The severity of the underlying stenosis did not appear to predict the site of the infarct. Similarly, Ellis and colleagues found high-grade stenosis to be an uncommon cause of perioperative MI [18]. They also reported viable myocardial areas with collateralised total occlusions to be the predominant site of perioperative myocardial infarctions. The extent of coronary artery disease (CAD) in terms of the number of arteries involved as well as luminal irregularities in the coronary arteries correlated with the risk of MI [18].

## Role of preoperative coronary revascularisation

### Risk index stratification and symptom status in guiding management

Compared with the 2007 ACC/AHA guidelines which heavily relied on the risk of surgery in determining the need for further testing in CAD patients with poor functional capacity, the 2014 guidelines incorporate both surgical and clinical risk using NSQIP and RCRI risk models [26]. It might be preferable to utilise both these risk models in a given patient, as RCRI alone may not perform well in predicting perioperative events in patients undergoing vascular surgery [27].

### Impact of prior revascularisation

For patients with stable asymptomatic CAD who are not at high risk for perioperative cardiovascular events, current ESC guidelines do not recommend routine angiography if patients have undergone CABG in the past six years based on data from Coronary Artery Surgery Study (CASS) [2, 3]. The retrospective analysis of results from the CASS database involving 25,000 patients demonstrated that the protective effect of CABG is more prominent in patients with triple-vessel disease and/or low left ventricular ejection fraction for at least six years. Of note, these 3368 operations included a mix of abdominal, thoracic, vascular and head and neck surgeries.

### Routine coronary angiography approach

Coronary Artery Revascularisation Prophylaxis (CARP), which is the largest randomised trial to date, failed to show a benefit for routine coronary revascularisation when compared with medical management alone in reducing perioperative or long-term mortality [28]. In contrast, a more recent but smaller randomised controlled trial reported by Monaco and colleagues revealed startlingly different results, favouring a more systematic approach utilising routine preoperative coronary angiography and revascularisation in an effort to reduce major adverse events in the perioperative period as well as better long-term survival compared with stress-test based strategy (Table 1; [11]).

**Table 1:** Major trials comparing coronary revascularisation versus no coronary revascularisation preoperatively

Study (Year)	*N*	Type of surgery	Strategy (*n*)	LM (%)	3vCAD (%)	1–2 VD (%)	Revasc mode (%)		Outcome	
							CABG	PCI	30-day MACE	Long-term mortality
CARP 2004 [24]	510	AAA, Vasc	CR (*n* = 258)	0	35.3	64.7	41	59	No difference in death or PMI	22 % (+REV) vs 23 % (−REV) at 2.7 years (*p* = 0.92)
			No CR (*n* = 252)	0	31.3	68.7	0	0		
Monaco 2009 [3]	208	AAA, Vasc	CA+CR (*n* = 61) *n* = 105^a^	13.1	44.3	55.8	47.5	52.5	4.8 % vs 11.7 % (*p* = 0.1)	Better survival in CA + CR at 5 years (*p* = 0.01)
			ST-based CR (*n* = 41) *n* = 103^a^	9.5	38.1	56.9	28.6	71.4		
Illuminati 2010 [22]	426	CEA	CA+CR (*n* = 216)	4	4	92	3	97	Mortality: no difference in PMI: CA + CR better (*p* = 0.01)	*N*/A
			No CA/CR (*n* = 210)	*N*/A	*N*/A	*N*/A	0	0		

*CA* coronary angiography, *CR* coronary revascularisation, *AAA* abdominal aortic aneurysm, *Vasc* vascular, *CEA* carotid endarterectomy, *LM* left main, *3vCAD* three vessel coronary artery disease, *VD* vessel disease, *Revasc* revascularisation, *CABG* coronary artery bypass grafting, *PC* percutaneous coronary intervention, *REV* revascularisation, *PMI* perioperative non-fatal myocardial infarction, *MACE* major adverse cardiac events, *ST* stress test

^a^Total patients randomised to each group

The CARP trial screened 5859 patients across 18 Veterans Affairs (VA) hospitals scheduled for elective vascular surgery [28]. All patients underwent routine coronary angiography based on either clinical assessment or non-invasive stress testing. Patients found to have >70 % coronary stenosis were randomised to revascularisation and medical management versus medical management only. Patients with significant left main disease, severe aortic stenosis and those with left ventricular ejection fraction <20 % were excluded. While only 9 % of the original cohort (510 patients) were randomised, only 32 % of the randomised patients had triple-vessel disease, and only 44 % had moderately large areas of ischaemia on nuclear stress imaging. Approximately 60 % of the patients were asymptomatic from a coronary standpoint. The study reported no benefit of prophylactic coronary revascularisation in reducing perioperative or long-term mortality. However, the CARP trial had limitations. By excluding patients who are likely to have derived most benefit from revascularisation, what the study failed to ascertain was whether patients with more extensive CAD would actually benefit from prophylactic revascularisation or not in the perioperative setting. Additionally, patients were selected for angiography only if they had risk factors or an abnormal stress test, which precludes detection of significant asymptomatic CAD in these high-risk patients undergoing vascular surgery. Since atherosclerosis is the common pathogenic mechanism in patients with CAD and peripheral arterial disease, these patients are quite likely to have significant disease in other vascular beds. For instance, incidence of severe CAD in patients with carotid artery disease can be as high as 72 % [29]. Another study on patients undergoing revascularisation of lower extremity peripheral artery disease demonstrated a 66 % incidence of concomitant significant CAD [30]. A subgroup analysis of 109 patients from the CARP trial who underwent surgery for abdominal aortic aneurysm (AAA) and had an abnormal preoperative stress test, prophylactic coronary revascularisation was associated with a reduced risk of death and nonfatal MI. Besides, ischaemia of the anterior myocardium was a predictor of poor outcomes independent of revascularisation [31].

In contrast to the CARP trial, two relatively recent trials have tested a strategy of routine coronary angiography followed by revascularisation as needed regardless of symptomatic status, which demonstrated significant reduction in cardiac adverse events perioperatively [11, 12]. In a trial involving patients scheduled for carotid endarterectomy, patients were randomised to routine coronary angiography versus none in patients without any history or symptoms of CAD. The results demonstrated that routine coronary angiography followed by prophylactic coronary revascularisation reduced the incidence of postoperative cardiac ischaemic events [12]. Similarly, an older study on patients undergoing open repair of AAA showed improved outcomes with routine coronary angiography [7].

Monaco and colleagues randomised 208 patients scheduled for elective AAA repair [11]. By using the RCRI, patients with ≥2 risk factors were randomly assigned to either a selective or a systematic strategy. The patients in the selective strategy group underwent non-invasive stress testing first followed by coronary angiography only if a preceding stress test was abnormal; approximately 41 % of patients in this group underwent revascularisation. In the systematic strategy group, all patients underwent coronary angiography without a preceding stress test; approximately 58 % of the patients in this group underwent revascularisation. Almost all patients in both groups underwent complete revascularisation. At a follow-up of almost five years, patients in the systematic group had improved survival (*p* = 0.01), as well as freedom from major adverse cardiovascular events (*p* = 0.003). This study highlights two potential points. Firstly, it did not use the patients’ functional capacity in determining stress testing vs coronary angiography, contrary to the current ACC/AHA guideline recommendations. Secondly, it reinforces the approach that patients with evidence of clinically significant atherosclerosis elsewhere may benefit from direct angiography in detecting significant CAD without prior stress testing. The fact that routine coronary angiography showed better survival when compared with prior stress testing may be explained in theory by two potential reasons. Firstly, the risk of a false-negative stress test is abolished; of note, underestimation of CAD in these patients, possibly due to ‘balanced’ ischaemia, has been reported with the use of non-invasive testing [32]. Secondly, the pathophysiological mechanism of perioperative myocardial ischaemia could be entirely different from that of the stress induced during the stress testing, as explained above.

### Stress-based approach

In a retrospective analysis, Landesberg et al. found that patients who had moderate-to-severe ischaemia on preoperative thallium perfusion scanning and underwent coronary revascularisation had better long-term survival when compared with patients who did not undergo revascularisation [9]. In contrast to the CARP trial where only one-third of the enrolled patients had three-vessel CAD and all patients with left main disease were excluded, 73 % of patients in this retrospective trial had left main or triple-vessel CAD. In addition, it also compared patients with normal, mild, and moderately severe but fixed perfusion deficits and concluded that results on preoperative thallium scanning can predict perioperative mortality. On the other hand, all patients undergoing major vascular surgery underwent thallium scanning as a routine, rather than risk stratification and assessment of functional capacity. Age, type of vascular surgery, previous MI and presence of diabetes were independent predictors of mortality. In another retrospective study by the same group, patients with abnormal myocardial perfusion studies undergoing major vascular surgery were divided into low, intermediate and high-risk categories based on risk factors (age, diabetes, cerebrovascular disease, ischaemic heart disease, congestive heart failure, ST depression on pre-op ECG and renal insufficiency) [10]. As expected, patients in the intermediate and high-risk groups had worse survival; however, preoperative coronary revascularisation following an abnormal thallium scan improved long-term survival in the intermediate risk group only.

Recently, a retrospective analysis on 1104 patients was completed. It demonstrated that when compared with patients with non-revascularised CAD, patients who had undergone coronary revascularisation had a significant improvement in perioperative cardiac mortality [33]. However, overall mortality did not change with coronary revascularisation. This was mainly attributed to non-cardiac peripheral vascular adverse events.

## Discussion

In summary, the pathophysiology of perioperative MI is complex, and is attributable to both a primary coronary plaque disruption as well as a demand-supply mismatch triggered by the haemodynamic effects of surgery owing to a high catecholamine state. In patients with stable CAD and excellent exercise tolerance (>10 METS), current guidelines do not recommend further testing, while those with low or unknown functional capacity should undergo pharmacological testing if it will change the management in case of elective surgery. The jury is still out for patients with a moderate functional capacity (4-10 METS) [1].

What remains unclear is how the stress of surgery itself correlates with the described METS used to gauge functional capacity, given the varied mechanisms involved in perioperative myocardial ischaemia. Although functional capacity may predict the extent of oxygen demand-supply mismatch inducible by physical activity, what it fails to predict is the pro-thrombotic and pro-inflammatory changes in the perioperative period, which may contribute to cardiac ischaemic events. It is difficult to quantify the ‘stress’ that these factors may exert on the coronary vasculature and hence using functional capacity measurements by exercise capacity alone may be inadequate in predicting perioperative outcomes. In addition, the role of functional assessment of CAD by induced stress vs anatomical assessment using coronary calcium scoring has also been recently described. Patients with calcified atherosclerotic plaques even in the absence of inducible perfusion defects have shown an increased risk for adverse cardiovascular events [34]. It may be argued that atherosclerosis is a progressive process with multiple organ involvement; hence patients with significant atherosclerotic disease elsewhere, even in the absence of clinical symptoms of CAD, may actually have significant disease. Given these limitations of stress testing, preoperative angiography in high-risk patients (RCRI >3) undergoing elevated-risk surgery (NSQIP risk of MI and cardiac arrest >1 %) may be beneficial [35].

For patients determined to be at a low risk for major adverse cardiac events (NSQIP risk <1 %), it may be reasonable to proceed with surgery without stress testing and/or coronary angiography, with optimisation of medical therapy. On the other hand, in patients deemed high risk, especially those with poor functional capacity, future studies will need to address whether a selective strategy using a stress test first versus routine coronary angiography guided management could provide a more desirable perioperative outcome of low incidence of MACE. The next question is that based on coronary angiography, which patients would benefit from revascularisation: only those with multi-vessel disease, those with proximal left anterior descending artery or those requiring coronary bypass grafting. The answers to these questions remain debatable, hence the level of evidence is C (expert opinion) in the most recent ACC/AHA guideline when it comes to preoperative coronary revascularisation calling for further studies. What is interesting is that the latest ACC/AHA guidelines did not incorporate the data from relatively recent trials where routine coronary angiography was utilised [11, 12].

Nevertheless, based on the literature reviewed, the authors of this review would lean toward a lower threshold routine angiography and revascularisation as clinically deemed appropriate for high-risk patients undergoing high-risk surgery. In this scenario, although prior stress testing might be helpful, lack of inducible ischaemia may lead to underestimation of the potential risk and possible benefit of coronary revascularisation. Even though the primary endpoint was negative, patients undergoing ‘complete’ revascularisation derived perioperative benefit in the CARP trial [31]. For patients determined to be at intermediate risk, however, it may be beneficial to conduct stress testing followed by angiography and revascularisation if the test is moderately or severely abnormal. Inarguably, further large randomised controlled trials comparing routine coronary angiography versus the current ACC/AHA and ESC guidelines would help in caring for these complex patient subsets.

## References

[1] Fleisher LA, Fleischmann KE, Auerbach AD (2014). ACC/AHA Guideline on Perioperative Cardiovascular Evaluation and Management of Patients Undergoing Noncardiac Surgery: A Report of the American College of Cardiology/American Heart Association Task Force on Practice Guidelines. J Am Coll Cardiol.

[2] Kristensen SD, Knuuti J, Saraste A (2014). ESC/ESA Guidelines on non-cardiac surgery: cardiovascular assessment and management: The Joint Task Force on non-cardiac surgery: cardiovascular assessment and management of the European Society of Cardiology (ESC) and the European Society of Anaesthesiology (ESA). Eur Heart J.

[3] Eagle KA, Rihal CS, Mickel MC (1997). Cardiac risk of noncardiac surgery: Influence of coronary disease and type of surgery in 3368 operations. Circulation.

[4] Hertzer NR, Young JR, Beven EG (1987). Late results of coronary bypass in patients presenting with lower extremity Ischemia: the Cleveland clinic study. Ann Vasc Surg.

[5] Hertzer NR, Young JR, Kramer JR (1979). Routine coronary angiography prior to elective aortic reconstruction: results of selective myocardial revascularization in patients with peripheral vascular disease. Arch Surg.

[6] Beven EG (1986). Routine coronary angiography in patients undergoing surgery for abdominal aortic aneurysm and lower extremity occlusive disease. J Vasc Surg.

[7] Bayazit M, Göl MK, Battaloglu B, Tokmakoglu H, Tasdemir O, Bayazit K (1995). Routine coronary arteriography before abdominal aortic aneurysm repair. Am J Surg.

[8] Hertzer HR, Beven EG, Young JR (1984). Coronary artery disease in peripheral vascular patients. A classification of 1000 coronary angiograms and results of surgical management. Ann Surg.

[9] Landesberg G, Mosseri M, Wolf Y (2003). Preoperative thallium scanning, selective coronary revascularization, and long-term survival after major vascular surgery. Circulation.

[10] Landesberg G, Berlatzky Y, Bocher M (2007). A clinical survival score predicts the likelihood to benefit from preoperative thallium scanning and coronary revascularization before major vascular surgery. Eur Heart J.

[11] Monaco M, Stassano P, di Tommaso L (2009). Systematic strategy of prophylactic coronary angiography improves long-term outcome after major vascular surgery in medium- to high-risk patients: a prospective, randomized study. J Am Coll Cardiol.

[12] Illuminati G, Ricco JB, Greco C (2010). Systematic preoperative coronary angiography and stenting improves postoperative results of carotid endarterectomy in patients with asymptomatic coronary artery disease: a randomised controlled trial. Eur J Vasc Endovasc Surg.

[13] Pierpont GL, Moritz TE, Goldman S (2004). Disparate opinions regarding indications for coronary artery revascularization before elective vascular surgery. Current opinion on revascularization study investigators. Am J Cardiol.

[14] Priebe HJ (2004). Triggers of perioperative myocardial ischaemia and infarction. Br J Anaesth.

[15] Landesberg G (2003). The pathophysiology of perioperative myocardial infarction: facts and perspectives. J Cardiothorac Vasc Anesth.

[16] Mangano DT, Browner WS, Hollenberg M, London MJ, Tubau JF, Tateo IM (1990). Association of perioperative myocardial Ischemia with cardiac morbidity and mortality in men undergoing noncardiac surgery. N Engl J Med.

[17] Beattie WS, Buckley DN, Forrest JB (1993). Epidural morphine reduces the risk of postoperative myocardial ischaemia in patients with cardiac risk factors. Can J Anaesth.

[18] Ellis SG, Hertzer NR, Young JR, Brener S (1996). Angiographic correlates of cardiac death and myocardial infarction complicating major nonthoracic vascular surgery. Am J Cardiol.

[19] Landesberg G, Mosseri M, Zahger D (2001). Myocardial infarction after vascular surgery: the role of prolonged, stress-induced, ST depression-type ischemia. J Am Coll Cardiol.

[20] Bursi F, Babuin L, Barbieri A (2005). Vascular surgery patients: perioperative and long-term risk according to the ACC/AHA guidelines, the additive role of post-operative troponin elevation. Eur Heart J.

[21] Raby KE, Brull SJ, Timimi F (1999). The effect of heart rate control on myocardial ischemia among high-risk patients after vascular surgery. Anesth Analg.

[22] Landesburg G, Zhou W, Aversano T (1999). Tachycardia-induced subendocardial necrosis in acutely instrumented dogs with fixed coronary stenosis. Anesth Analg.

[23] Cohen MC, Aretz TH (1999). Histological analysis of coronary artery lesions in fatal postoperative myocardial infarction. Cardiovasc Pathol.

[24] Duvall WL, Sealove B, Pungoti C, Katz D, Moreno P, Kim M (2012). Angiographic investigation of the pathophysiology of perioperative myocardial infarction. Catheter Cardiovasc Interv.

[25] Dawood MM, Gutpa DK, Southern J, Walia A, Atkinson JB, Eagle KA (1996). Pathology of fatal perioperative myocardial infarction: implications regarding pathophysiology and prevention. Int J Cardiol.

[26] Oprea AD, Fontes ML, Onaitis MW, Kertai MD (2015). Comparison between the 2007 and 2014 American College of Cardiology/american heart association guidelines on perioperative evaluation for noncardiac surgery. J Cardiothorac Vasc Anesth.

[27] Ford MK, Beattie WS, Wijeysundera DN (2010). Systematic review: prediction of perioperative cardiac complications and mortality by the revised cardiac risk index. Ann Intern Med.

[28] McFalls EO, Ward HB, Moritz TE (2004). Coronary artery revascularization before elective major vascular surgery. N Engl J Med.

[29] Hur DJ, Kizilgul M, Aung WW, Roussillon KC, Keeley EC (2012). Frequency of coronary artery disease in patients undergoing peripheral artery disease surgery. Am J Cardiol.

[30] Lee MS, Rha SW, Han SK (2015). Clinical outcomes of patients with critical limb ischemia who undergo routine coronary angiography and subsequent percutaneous coronary intervention. J Inv Cardiol.

[31] Garcia S, Rider JE, Moritz TE (2011). Preoperative coronary artery revascularization and long-term outcomes following abdominal aortic vascular surgery in patients with abnormal myocardial perfusion scans: a subgroup analysis of the coronary artery revascularization prophylaxis trial. Catheter Cardiovasc Interv.

[32] Berman DS, Kang X, Slomka PJ (2007). Underestimation of extent of ischemia by gated SPECT myocardial perfusion imaging in patients with left main coronary artery disease. J Nucl Cardiol.

[33] Ultee KHJ, Rouwet EV, Hoeks SE (2015). Coronary revascularization induces a shift from cardiac toward noncardiac mortality without improving survival in vascular surgery patients. J Vasc Surg.

[34] Chang SM, Nabi F, Xu J (2009). The coronary artery calcium score and stress myocardial perfusion imaging provide independent and complementary prediction of cardiac risk. J Am Coll Cardiol.

[35] Landesberg G, Mosseri M (2009). Prophylactic Pre-Operative Coronary Revascularization: Is the Phoenix Awakening?. J Am Coll Cardiol.

